# Draft Genome Sequence of the Plant Growth-Promoting *Cupriavidus gilardii* Strain JZ4 Isolated from the Desert Plant *Tribulus terrestris*

**DOI:** 10.1128/genomeA.00678-16

**Published:** 2016-07-28

**Authors:** Feras F. Lafi, Ameerah Bokhari, Intikhab Alam, Vladimir B. Bajic, Heribert Hirt, Maged M. Saad

**Affiliations:** a King Abdullah University of Science and Technology (KAUST), Center for Desert Agriculture (CDA), Biological and Environmental Sciences and Engineering Division (BESE), Thuwal, Kingdom of Saudi Arabia; b King Abdullah University of Science and Technology (KAUST), Computational Bioscience Research Center (CBRC), Thuwal, Kingdom of Saudi Arabia

## Abstract

We isolated the plant endophytic bacterium *Cupriavidus gilardii* strain JZ4 from the roots of the desert plant *Tribulus terrestris*, collected from the Jizan region, Saudi Arabia. We report here the draft genome sequence of JZ4, together with several enzymes related to plant growth-promoting activity, environmental adaption, and antifungal activity.

## GENOME ANNOUNCEMENT

An endophytic bacterium, *Cupriavidus gilardii* strain JZ4, was isolated from the surface of sterilized roots of the desert pioneer plant *Tribulus terrestris*. Plants were collected from the Jizan region (16°56.475′N, 42°36.694′E) in Saudi Arabia. The JZ4 strain shows plant growth-promoting activity *in vitro* when tested with *Arabidopsis thaliana*. Based on the 16S rRNA sequence similarity, the JZ4 strain was closely related (99% similarity) to *C. gilardii* CR3 (1). The JZ4 strain was also related to other *Cupriavidus* spp. such as *C. metallidurans* CH34 (2), which is found exclusively in heavy metal-contaminated environments (3–5). The JZ4 strain is also closely related to plant-associated species such as the nitrogen-fixing symbiont of legumes *C. taiwanensis* strain LMG19424 (6) and *C. plantarum* isolated from the rhizosphere of sorghum (7).

The genomic DNA was isolated using the Qiagen DNeasy blood and tissue kit following the instructions from the manufacturer. For genome sequencing, we used the Illumina TrueSeq sample preparation kit and, following the manufacturer’s protocol, generated a paired-end library that had a calculated average insert size of 500 bp. The Illumina MiSeq platform was used to generate DNA sequence reads of 300 bp and with a theoretical coverage of 100-fold. Adapter sequences were removed with Illumina’s built-in read trimming tool and through the Spades assembler version 3.6 (8). MegaBLAST searches of strain JZ4 concatenated genomes against the NCBI reference genomic sequence database revealed that the closest relative genome was *C. gilardii* CR3 (1).

Functional annotation of the JZ4 strain was performed using the INDIGO pipeline (9) with the exception of the prediction of open reading frames (ORFs) by FragGeneScan (10). The genome sequence has 3,940 ORFs, 7 rRNAs, 53 tRNAs, and 92 ncRNAs. The annotation revealed both isochorismate lyase (EC 4.2.99.21) and isochorismatase (EC 3.3.2.1), two enzymes that are involved in salicylic acid production (11). Moreover, the genome contains genes encoding cholesterol oxidase (EC 1.1.3.6), which have been developed as a biotechnological tool to infer insect resistance in plants (12), and chitinase enzyme (EC 3.2.1.14), which has been reported to be involved in fending off plant pathogenic fungi (13). Further analysis of the genome sequence of the JZ4 strain will help to elucidate the metabolic pathways involved in plant growth promotion and provide further insights into the molecular strategies to control soilborne plant diseases.

### Nucleotide sequence accession numbers.

The genome of *Cupriavidus gilardii* JZ4 was deposited at DDBJ/EMBL/GenBank under the accession number LVXY00000000. The version described in this paper is the first version, LVXY01000000.
